# Topographically Engineered Large Scale Nanostructures for Plasmonic Biosensing

**DOI:** 10.1038/srep24385

**Published:** 2016-04-13

**Authors:** Bo Xiao, Sangram K. Pradhan, Kevin C. Santiago, Gugu N. Rutherford, Aswini K. Pradhan

**Affiliations:** 1 Department of Engineering and Center for Materials Research, Norfolk State University, Norfolk, VA 23504, USA

## Abstract

We demonstrate that a nanostructured metal thin film can achieve enhanced transmission efficiency and sharp resonances and use a large-scale and high-throughput nanofabrication technique for the plasmonic structures. The fabrication technique combines the features of nanoimprint and soft lithography to topographically construct metal thin films with nanoscale patterns. Metal nanogratings developed using this method show significantly enhanced optical transmission (up to a one-order-of-magnitude enhancement) and sharp resonances with full width at half maximum (FWHM) of ~15nm in the zero-order transmission using an incoherent white light source. These nanostructures are sensitive to the surrounding environment, and the resonance can shift as the refractive index changes. We derive an analytical method using a spatial Fourier transformation to understand the enhancement phenomenon and the sensing mechanism. The use of real-time monitoring of protein-protein interactions in microfluidic cells integrated with these nanostructures is demonstrated to be effective for biosensing. The perpendicular transmission configuration and large-scale structures provide a feasible platform without sophisticated optical instrumentation to realize label-free surface plasmon resonance (SPR) sensing.

Surface plasmon resonance, the collective oscillation of electrons bound to a metallic surface, plays a critical role in the manipulation of light. The incident light may couple to the surface plasmon polariton (SPP) with properly arranged nanostructures if the wave vector satisfies the following general momentum-matching condition: 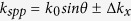, where *k*_*spp*_ is the surface plasmon wave vector, *k*_*0*_ is the component of the wave vector of the incident light, *θ* is the incident angle, and Δ*k*_*x*_ is from any perturbation across the interface^1^. Artificial and engineered nanostructures provide a suitable paradigm for the manipulation of such perturbations. As a general approach, periodically constructed nanostructures could excite plasmon resonances at a specific frequency, as the wave vectors meet the condition Δ*k*_*x*_ = *nk*_*g*_ with an integer *n* and *k*_*g*_ = *2π/p*, where *p* is the periodicity of the nanostructure, and *k*_*g*_ is the wave vector. The conventional SPR system fulfills the momentum-matching condition through the incident angle *θ*, which generally requires a rotary stage or goniometer to precisely detect the reflection angle and control the incident direction. Alternatively, the condition could be fulfilled by properly arranged nanostructures even for the normal incident light (*θ* = 0°). Such a configuration can significantly lower the instrumentation requirements and open up an exciting new avenue for applications in subwavelength optics, energy harvesting, imaging, chemical and biological sensing^2^,^3^,^4^,^5^,^6^.

Recently, light transmission through a subwavelength aperture or an array of apertures, such as nanoholes and nanoslits^7^,^8^,^9^,^10^ has been extensively studied. These studies have revealed the intricate manipulation of interactions between light and nanostructures. At the normal incident light, the enhanced resonant transmission is not a unique phenomenon in the perforated metal thin films. Corrugated metal films or flat metal films with periodically arranged nanostructures can excite plasmon resonances at one or both sides of the film, which results in similar transmission effects^11^,^12^,^13^,^14^. As reported in our previous work, nanostructured metal thin films demonstrated distinct asymmetric Fano resonances observed in the zero-order transmission spectra using an incoherent light source. The transmission efficiency can surpass that of a metal thin film with the same area and thickness at the resonance frequency by severalfold. Figure 1 shows the structural configuration, theoretical analysis and experimental results for this nanostructured metal thin film. Using the method developed previously, silver metal thin films covering SiO_2_ nanograting structures can yield enhanced optical transmission at the normal incidence using a broadband white light source^14^. Such structural mediated metal thin films achieved coherent surface plasmon excitation and resulted in sharp Fano resonances (Fig. 1b). In addition, the resonance wavelength can be controlled by the nanostructure periodicity.

A general interpretation of the phenomenon is represented by the well-accepted Bloch-mode excitation of a surface electromagnetic wave in the dielectric and metal interface. In these periodic structures, a theoretical approach can treat the electromagnetic fields as Fourier sums that meet Bloch’s boundary condition. Using the Rayleigh method, the electromagnetic field component is written in the sum of the form^9^:


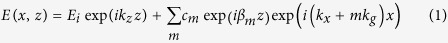


where the incident electromagnetic wave is propagating along the *z* direction normal to the interface planes and the periodic surface profile is along the *x* direction, *β*_*m*_ is the Bragg vectors, and *k*_*g*_ is the wave vector of the periodic structure. As the electromagnetic wave propagates along the *z* direction, the first term on the left side of the equation (1) decays rapidly through the metal thin film. The second term in Eq. 1 contributes largely to the distribution of the total transmitted field. Although the Bragg vector is independent of the nanostructure shape, a large number of the coefficients (*c*_*m*_) rely on the structure profile. It becomes difficult to derive the equation above for arbitrary structures due to the complexity of the profiles. However, it can be numerically analyzed to understand the physical properties. Here, we resolve the analysis in a single nanostructure instead of a periodic array to microscopically investigate the mechanism. We extend the basic mechanism at the microscopic level to present an intuitive picture for a physical interpretation of the phenomenon. Finite-difference-time-domain (FDTD) simulation is employed to study the spatial field distribution. For simplicity, the analysis is only focused on the normal incidence, where *k*_*x*_ = *0*. Moreover, the structure profile is extracted from the SEM images to closely match the fabricated samples. The radiant characteristic of the plasmonic mode is studied from the near-field characteristics of the evanescent wave at the air/metal interface. Figure 1c shows the magnetic field distribution for a half of the single nanostructure under TM-polarized incident light (electric field *x*-polarized). The standing field pattern indicates the excitation of the surface plasmon at the interfaces of the metal thin film. Localized and enhanced fields are more easily achieved in the vicinity of this tip-like nanostructure because the high curvature amplifies the external field. This effect is analogous to the field emission enhancement at tip-like structures, in which the local field *F* is enhanced by the generalized relation of *F* = *γF*_0_, where *γ* is the field enhancement factor and *F*_*0*_ is the global electric field^15^. In the plasmonic view, this nanostructure is simply considered to be a rough surface. The curved surface can yield an infinite number of radiative eigenmodes and couple them with photons^1^. Therefore, the high transmission efficiency is attributed partly to the enhanced field. However, this alone does not provide a sufficient explanation for the total enhancement and the wavelength dependence of the nanostructure array. To further understand the enhanced transmission, we perform the calculation of the field propagating through a single nanostructure and then extract the evanescent field over the air/metal interface. The total *H*_*y*_ field (Fig. 1d) indicates the plasmon polaritons travel along the interface. By comparing a single SPP-mode wave, the two waves can be readily analyzed side by side. The total field along the interface is not a single SPP mode. We perform a spatial Fourier transformation to isolate the main contribution. Figure 1e shows that the location of the spatial frequency of the total field matches that of the single SPP-mode. The spatial frequency distribution indicates the wavelength depends on the incidence light wavelength. In the periodic structure, those SPP modes meet the Bloch condition and constructively couple with each other.

The experimental and numerical results hold true in the visible and near-infrared spectral ranges. Engineering such metallic nanostructures provide a new way to selectively manipulate electromagnetic waves across the entire solar spectrum and beyond. We have used a high-throughput nanofabrication technique by combing features of nanoimprint and soft lithography to produce wafer-scale nanostructured patterns. The method is similar to the fabrication of gold nanodome arrays^16^. It transfers nanostructure patterns from a reusable master template to transparent substrates through a stamp. The major steps of our fabrication technique are shown in Fig. 2a. The master templates with nanostructure patterns can be produced by well-developed techniques, such as electron beam lithography (EBL), focus ion beam (FIB), and interference lithography (IL), etc. In our experiment, EBL is used to generate the nanostructure patterns on the silicon substrate. Chromium is deposited by electron beam evaporation on the patterned substrates and a lift-off process is followed to form the nanostructures. To achieve the smooth surface, we introduced an additional process in which the nanopatterned masters are coated with a thin polymer layer (Fig. S1).

This is a critical process to obtain smooth surfaces and achieve a high coupling efficiency on the final plasmonic structures. Theoretically, when light propagates through these nanostructure systems, the electromagnetic problem can be casted as a linear Hermitian eigenvalue problem with a close analogy to the quantum mechanical case. In reality, the validation of such a mathematical model requires the following conditions: (i) an individual nanostructure can effectively form a specific spatial distribution of the fields; and (ii) those fields can efficiently couple with each other. The disparities among nanoparticles cause a random distribution of scattered light in all directions. In artificial and engineered nanostructures, rough surfaces, sharp corners and edges are generally inherited from the fabrication processes, and their size, shape and distribution are random. Therefore, it is necessary to eliminate or alleviate sharp corners and edges to significantly improve field coupling efficiency. For this purpose, a thin polymethyl methacrylate (PMMA) layer is spin-coated on the master.

Next, a transparent elastomer (polydimethylsiloxane, PDMS) stamp is cast onto the master to duplicate the nanoscale features. Multiple PDMS stamps can be replicated from a single master, and all of these stamps can be reused for the fabrication. Figure 2b shows pictures of a silicon master, a PDMA stamp and the nanostructured sample patterned with 6 mm × 10 mm nanograting structures (*p* = 600 nm). Throughout the process, the production of the master is the only step involving time-consuming and costly procedures. However, the master template process is only required for the initial stamp production. The primary fabrication of the nanostructured metal thin films is a simple mechanical stamping and metal deposition process to transfer nanostructures onto a transpent support (moldable) layer. In our experiments, we chose epoxy-based SU-8 photoresist as our imprint resist or support layer due to its high optical transmittance and reliability as a permanent structure after curing. The pressure and temperature, as well as other parameters, depend on the imprint resist layer. Our imprint temperature is set at 90 °C. The stamp is brought into contact with SU-8 coated glass slides under a weight of ~200 g (including the stamp’s weight) pressing for 2 min. The stamped samples are then exposed by a broadband mask aligner to harden or cure the SU-8 photoresist. Finally, a 45 nm Au or Ag metal thin film is deposited on the patterned substrates by electron beam evaporation. This simple process replicates the nanopatterns from the master to the desired substrates. More importantly, the final metal thin film nanostructures achieve significantly improved surface smoothness because of the thin layer coating on the master. AFM images revealed that sharp edges, pikes and corners are significantly alleviated (Fig. 2c). Figure 2d–g show the SEM images of several different nanostructures made by this technique. In addition to nanogratings (Fig. 2e), the technique is also able to generate other commonly studied nanostructures, such as the bullseye and nanodot (Fig. 2f,g) for plasmonics and metamaterials^2^,^8^,^17^,^18^.

This fabrication technique is a rapid way to reproduce large amounts of nanostructured chip devices. In addition, the large-scale nanostructures on the single chip allow us to perform optical characterization using conventional spectrophotometer systems. Unusually high transmission peaks with a distinct asymmetric Fano resonance line shape were identified from the transmission spectrum. As shown in Fig. 3a, nanostructures with the period of 600 nm were investigated, and the resonance wavelength was *λ* ≈ *p* (where *p* is the period). The transmission efficiencies observed at the resonance maxima are up to nearly one order of magnitude higher over those of the thin film with the same fraction and thickness. Strong coherent responses were revealed as the sharp resonance peaks, the FWHM of which is ~15 nm, which is limited by our EBL stitching accuracy. (The nanostructures developed from a single write field of EBL without stitching can achieve FWHMs below 10 nm.) The transmission spectra exhibited enhanced or extraordinary optical transmission (EOT) similar to that observed for subwavelength nanohole arrays^7^,^19^,^20^,^21^,^22^. Furthermore, the sharp resonance is a manifest of the coherent coupling the plasmonic oscillations, which are sensitive to the refractive index. Therefore, this plasmonic nanostructured thin film is a promising platform for SPR sensor devices. Most importantly, because the plasmonic momentum matching is modulated by the nanostructures, the signal input and detection are set up in a one-axis configuration in which a detection system would eliminate the requirement for sophisticated instrumentation. White light or natural light could be used as the incident source, and a conventional spectrophotometer is capable of performing the detection. (The transmission experiments and biosensing tests in Figs 3a,b and 4a are performed by a regular spectrophotometer, Perkin-Elmer Lambda 800, without any modification.) The refractive-index sensitivity is the basis of plasmonic detection. Figure 3b demonstrates this concept to determine the spectral shift using NaCl solutions of various concentrations (5%, 10%, 15% and 20%) in deionized water, corresponding to refractive indices of 1.3418, 1.3505, 1.3594, and 1.3684, respectively^23^. The measurements were performed under TM-polarized light at normal incidence. A general sensitivity figure, *S* = Δ*λ/*Δ*n (nm RIU*^−*1*^) was used to quantify the performance of the plasmonic nanostructure. For the nanogratings with a 600 nm pitch size, the nanostructures achieved a high sensitivity at ~600 *nm RIU*^−*1*^. Because we use the normal transmission for the sensing experiments, this spectral sensitivity number reaches the upper limit of the single-mode SPR spectral sensitivity. Sensitivity over this limit is beyond the scope of this letter. However, such sensitivities can be achieved by introducing incident or detection angles^24^. A general figure of merit, *FOM* = *S (nm RIU*^−*1*^*)/Γ (nm)*, can also be used to quantify the performance of the plasmonic nanostructure in units of wavelength, where *S* is the sensitivity to refractive index and *Γ* is the resonance linewidth or FWHM. The sharp resonance peaks significantly improve the sensing performance. The measured FOMs are ~40 for the large scale nanostructures (6 mm × 10 mm). These FOMs are limited by our EBL stitching accuracy. The FOMs of the small scale nanostructures (80 μm × 80 μm in a single write field of EBL without stitching) can reach 80–130 reproducibly using the same fabrication method.

To interpret the sensing ability of these nanostructures, an analysis similar to that discussed above can be used to study the spatial Fourier frequency. We extract the evanescent field from the FDTD calculation in a single nanostructure, where the nanostructure is covered by a medium (refractive index *n* > *1*). The evanescent magnetic field *H*_*y*_ is plotted in Fig. 3c as a function of the incident wavelength (*λ*_*0*_) along the *x* coordinate. We also introduce two SPP modes with different wavelengths (*λ*_*0*_ and *λ*_*0*_/n) for comparison. In the spatial frequency spectra (Fig. 3d), the peak position of the spatial frequency of the total field is positioned at the location of the single SPP mode with the wavelength (*λ*_*0*_*/n*). These calculation results are consistent with the findings shown in Fig. 3b, where the wavelength shift is approximately (*n-1)g* (*g* is the periodicity of the nanostructures). In the medium, that the phase velocity *υ* is defined by *υ* = *c/n*, where *n* is the refractive index of the medium, and *c* is the speed of light in a vacuum (*n* = *1*). From a macroscopic perspective, the surface electromagnetic wave with matched phases is excited in the vicinity of nanostructures; their contributions to a constructive spatial field distribution are determined by the surrounding medium. The coherent coupling of the nanostructures in the Bloch mode requires matched SPP phases. The phase delay in the medium meets the matching condition, where the incident light wavelength is approximately the value of the *n* times of the nanostructure periodicity (*λ*_*0*_ *~* *ng*). Therefore, when the white light is incident through the sensing platform, the resonance wavelength directly corresponds to the refractive index of the medium in contact with the nanostructure surface, and any changes of the medium (refractive index) result in the wavelength shift of the resonance.

To determine the utility of the nanostructures for practical biosensing applications, we verified the behavior of the transmission spectra to measure molecular binding interactions. Figure 4a shows an end-point measurement acquired by the spectrometer to detect the protein capturing functionality. High-affinity protein A/G - IgG binding pairs were chosen in our experiment as a standard model for plasmonic biosensing characterization^25^,^26^,^27^. In the detection, the sensor area is applied at first with 0.5 mg/mL reagent protein A/G in phosphate-buffered saline (PBS) to capture protein IgG. Protein A/G is immobilized on the gold surface by physisorption. Sequentially, both proteins are applied to the nanostructure chip surface and incubated for 1 h at room temperature. At every step, unbounded protein is rinsed with PBS solution and DI water and then blow-dried with nitrogen gas. The peak of the resonance in the non-functionalized sensors is located at 598 nm as a reference, which matches the periodicity of the nanostructures. After functionalizing protein A/G, the peak position shifts to 606 nm. Due to the high affinity of protein A/G to the Fc regions of IgG, IgG (0.5 mg/mL in PBS) spotted on the sensor can be captured on the protein A/G functionalized surface. The final result showed a large resonance shift to 617 nm. A 19 nm red shift was detected in the transmission spectrum. We further conducted experiments on real-time monitoring of protein-protein binding interactions using a microfluidic cell in a modified microscope imaging system equipped with a white light source and a cooled CCD detector with a 0.2 nm resolution and a sampling rate of 0.1 Hz in a 40 nm wavelength scanning range. In the measurement, the protein A/G and IgG were diluted in 10 mM PBS buffer solution. Immobilization of the protein A/G was accomplished in only one injection step for surface activation. The sensor chip surface was activated for 1 h at a flow rate of 30 μL/min for 0.5 mg/mL protein A/G. After the activation process, the microfluidic cell was flushed with PBS buffer solution to remove the unattached protein A/G. Injection of 10 μg/mL IgG followed at the flow rate of 30 μL/min. Fig. 4b clearly shows signal changes corresponding to a dynamic equilibrium of the binding process as the analytes are injected. This test platform revealed a 2 nm peak shift for the low concentration of IgG.

Our results demonstrate that these nanostructured metal thin films can be easily fabricated and set up with regular spectrophotometers for plasmonic chemical and biological sensing applications. Depending on the nanostructural arrangement, the resonance frequency can be manipulated by varying the periodicity of the structure. The simplicity and flexibility of these structures and their fabrication methods promise new routes for the development of a nanostructured platform for lab-on-a-chip sensing devices and can be applied to or integrated into the conventional spectrophotometer-based systems for multifunctional analysis. The unique properties also give the engineered plasmonic nanostructures great advantages for use in energy harvesting, imaging and optical processing.

## Additional Information

**How to cite this article**: Xiao, B. *et al.* Topographically Engineered Large Scale Nanostructures for Plasmonic Biosensing. *Sci. Rep.* **6**, 24385; doi: 10.1038/srep24385 (2016).

## Supplementary Material

Supplementary Information

## Figures and Tables

**Figure 1:**
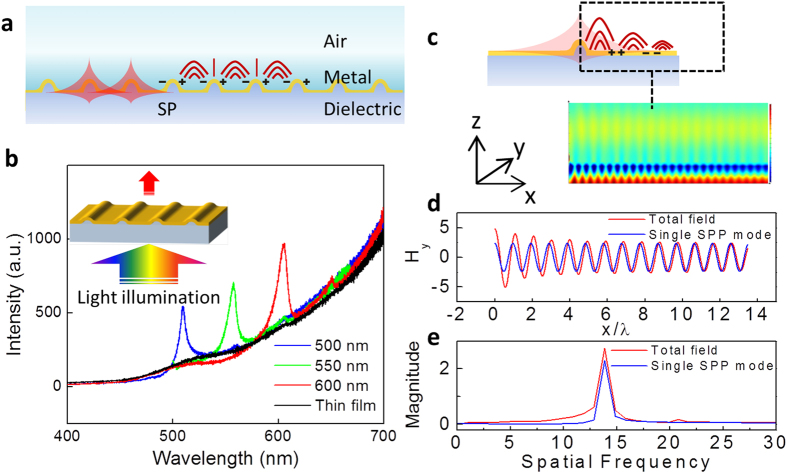
(**a**) Schematic of the nanostructured metal thin film and the SPs at the interface of the metal thin film and air. (**b**) Experimental optical transmission intensities of metal thin-film nanogratings under TM-polarized illumination for grating periods of 500, 550, and 600 nm. (**c**) 2-dimensional map of the real part of the total H_y_-component field in the x-z plane from FDTD simulation. (**d**) The magnitude of the total H_y_ component field and a single SPP-mode wave. (**e**) The spatial frequency spectra of the total field and single SPP.

**Figure 2:**
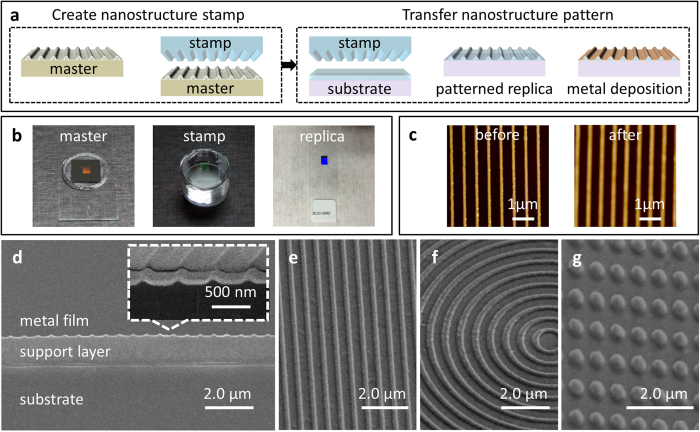
(**a**) Fabrication process flow diagram for nanostructured plasmonic metal thin films. (**b**) Pictures of an EBL patterned master on the silicon substrate, a PDMA stamp and a nanostructure replica with a gold thin film layer on a glass slide. (**c**) AFM images show the surface morphology of an EBL pattern master (before) and a stamped replica (after) (**d**). SEM images of a cross section of the structure. The nanostructure array has a period of 600 nm and a height of ~100 nm. The top metal layer is approximately 45 nm thick. (**e**) A nanograting structure. (**f**) A bullseye structure. (**g**) A silver array of ~250-nm-diameter nanodots spaced every 600 nm.

**Figure 3:**
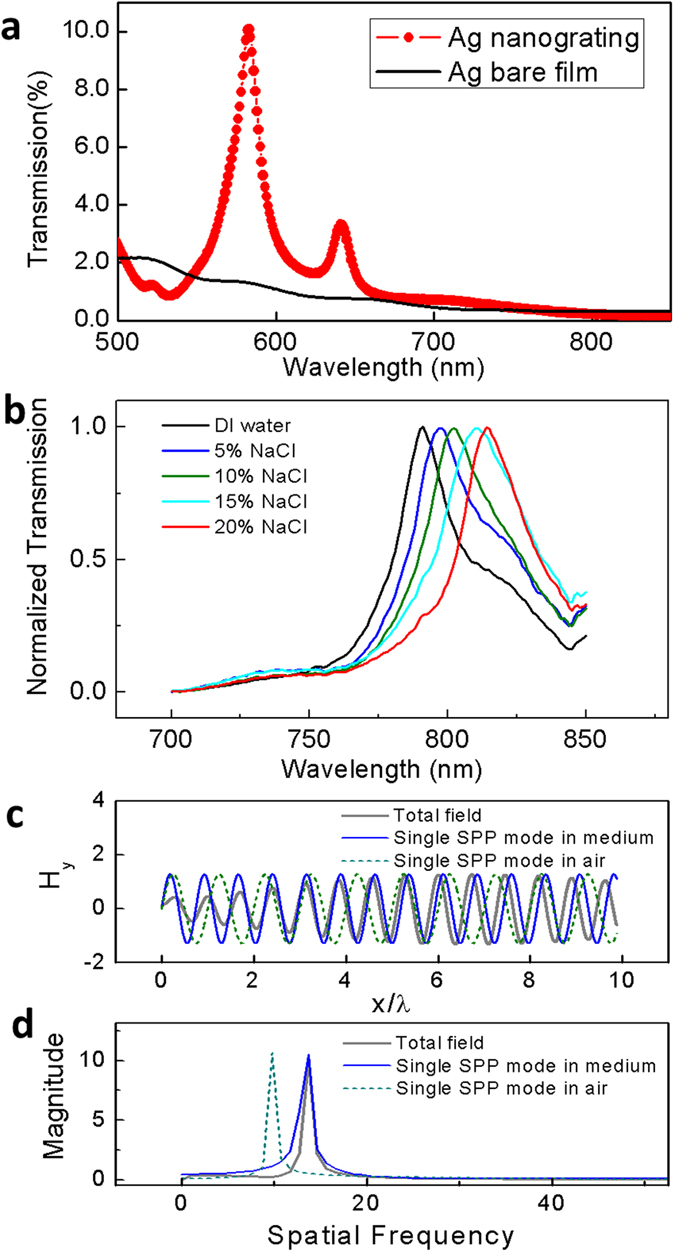
Transmission spectra through a nanostructured metal thin film and sharp resonance peak shift for biomolecular sensing. (**a**) Experimental optical transmission spectra of silver thin-film nanogratings under TM-polarized illumination for a grating period of 600 nm. 50 nm Ag nanostructured thin film (red) and the 50 nm Ag flat thin film (black). (**b**) Transmission spectra for a nanostructured gold thin film in DI water and NaCl solutions of various concentrations. The periodicity of the grating was 600 nm. (**c**) The magnitude of the total H_y_ component field and single SPP-mode waves in air and medium. (**d**) The spatial frequency spectra of the total field and single SPP waves in air and medium.

**Figure 4:**
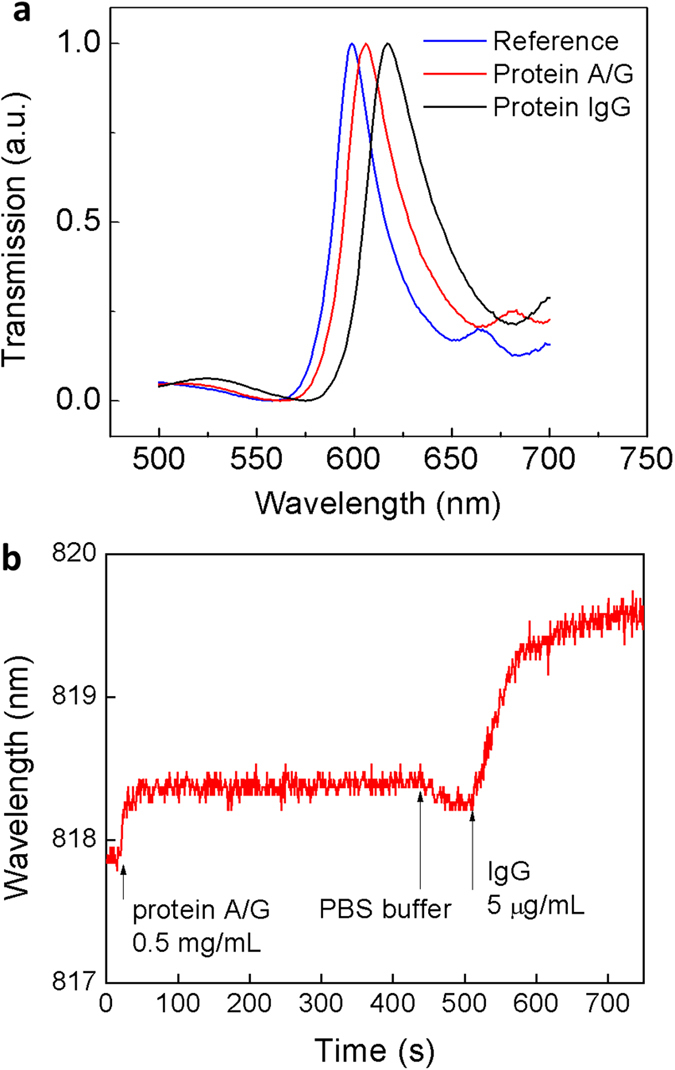
Gold nanostructured thin film sensors for observing the biomolecular binding reactions. (**a**) Transmission spectra of the bare sample as the reference, protein A/G physisorption and the capturing of the antibody (IgG). 45 nm Au nanostructured thin film with a period of 600 nm. (**b**) Response of the sensor device to the protein binding reaction of protein A/G and IgG. Sampling rate of 0.1 Hz at a scanning wavelength range of 800–840 nm.
