# Supplementary material for: Topographically Engineered Large Scale Nanostructures for Plasmonic Biosensing
Source: Sci Rep. 2016 Apr 13;6:24385. doi: 10.1038/srep24385 (PMC4829874; doi:10.1038/srep24385)
Supplement: Supplementary Information [file srep24385-s1.pdf]

Topographically Engineered Large Scale Nanostructures for Plasmonic Biosensing

Bo Xiao,* Sangram K. Pradhan, Kevin C. Santiago, Gugu N. Rutherford, Aswini K. Pradhan

Department of Engineering and Center for Materials Research, Norfolk State University, Norfolk, VA 23504, USA

*To whom correspondence should be addressed: bxiao@nsu.edu

Supporting Material

Master Fabrication Method

We have developed a simple process to obtain ultrasmooth nanostructures through the master fabrication process. Our master fabrication process is shown in Figure S1. Nanostructure arrays are fabricated on silicon substrates using electron-beam lithography. After the electron beam exposure and the developing of the resist, a 100 nm chromium layer was deposited on the substrate by electron beam evaporation. The critical step is the spin-coating of a thin layer (1% PMMA 950 in MIBK) onto the patterned substrate followed by the thermal baking of the substrate at 170 °C for 15 min. The coating and baking of the polymer produces a smooth curved layer around the nanostructures that determines the final topography. This process rounds the corners and edges and significantly contributes to the high reliability and reproducibility for the fabrication and the high coupling efficiency for the optical performance.

.

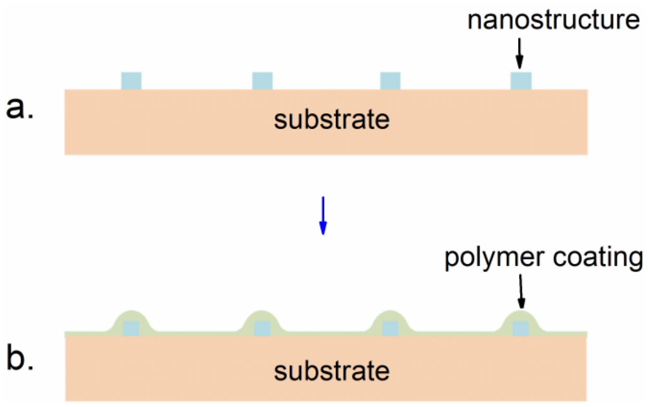

Figure S1. Master fabrication process: a. Nanostructure array patterning; b. Polymer layer preparing.

As shown in Figure S1.b, a thin polymer coating process has been introduced in our fabrication to smoothen the nanostructure surfaces in the fabrication of the masters. It’s not a trivial step. An ultrasmooth metal nanostructure can avoid unwanted roughness and impurities that seriously

degrade plasmonic performance. Our development can achieve such ultrasmoothness in the interfaces and significantly improved smoothness of the top surfaces. In addition, the thin polymer layer serves as a release layer during the step of separating cured PDMS stamps from the master substrates. Especially for large scale nanostructure replication, this layer can prevent the adhesion of PDMS on the rough nanostructures and avoid damaging nanostructures or master substrates.

Thickness Dependence of the Optical Transmission

These nanostructured thin films are designed to avoid sharp edges and corners. The fabrication process is simple. However, there are intricate variations involved in each fabrication step that introduce rich features into the final structures. Here, we demonstrate the thickness dependence of the nanostructured thin films. In general, the transmission efficiency of a metal thin film will decrease as the film thickness increases. The corresponding resonances of our nanostructured thin films show thickness dependence as shown in Fig. S2. From a simple viewport of scattering and diffraction, it would help to understand this physical phenomenon of the interaction of light and surface plasmon. The effect of the enhanced transmission can be considered as a constructive interference from the the air/metal and metal/medium interfaces instead of the destructive interference in Kretschmann configuration. If the metal film is too thin, it generally causes wide resonances due to the strong broad-band transmission (or considered as background noise). Thick metal, however, can reduce the interference between the interfaces. This dependence resembles to the Kretschmann configuration. The optimal thickness is similar to the conventional SPR around ~50 nm for gold and silver thin films.

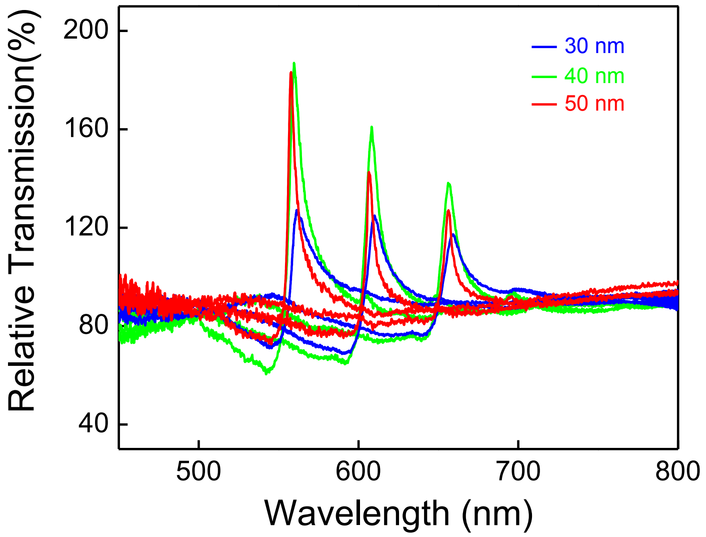

Figure S2. Transmission spectra of the thickness dependence of the transmission in the silver nanostructured thin films.
